# A randomized controlled intervention of workplace-based group cognitive behavioral therapy for insomnia

**DOI:** 10.1007/s00420-018-1291-x

**Published:** 2018-01-31

**Authors:** Helena Schiller, Marie Söderström, Mats Lekander, Kristiina Rajaleid, Göran Kecklund

**Affiliations:** 1 Stress Research Institute, Stockholm University, 10691 Stockholm, Sweden; 2 Department of Clinical Neuroscience, Karolinska Institutet, Stockholm, Sweden; 3 Center for Health Equity Studies, Stockholm University/Karolinska Institutet, Stockholm, Sweden; 4 KBT-Centralen, Stockholm, Sweden; 5 Behavioural Science Institute, Radboud University, Nijmegen, The Netherlands

**Keywords:** Group CBT, Insomnia, Sleep problem, Organizational intervention, Burnout, Chronic stress

## Abstract

**Purpose:**

Sleep disturbance is common in the working population, often associated with work stress, health complaints and impaired work performance. This study evaluated a group intervention at work, based on cognitive behavioral therapy (CBT) for insomnia, and the moderating effects of burnout scores at baseline.

**Methods:**

This is a randomized controlled intervention with a waiting list control group. Participants were employees working at least 75% of full time, reporting self-perceived regular sleep problems. Data were collected at baseline, post-intervention and at a 3-month follow-up through diaries, wrist-actigraphy and questionnaires including the Insomnia Severity Index (ISI) and the Shirom–Melamed Burnout Questionnaire (SMBQ). Fifty-one participants (63% women) completed data collections.

**Results:**

A multilevel mixed model showed no significant differences between groups for sleep over time, while there was a significant effect on insomnia symptoms when excluding participants working shifts (*N* = 11) from the analysis (*p* = 0.044). Moreover, a moderating effect of baseline-levels of burnout scores was observed on insomnia symptoms (*p* = 0.009). A post-hoc analysis showed that individuals in the intervention group with low burnout scores at baseline (SMBQ < 3.75) displayed significantly reduced ISI scores at follow-up, compared to individuals with high burnout scores at baseline (*p* = 0.005).

**Conclusions:**

Group CBT for insomnia given at the workplace did not reduce sleep problems looking at the group as a whole, while it was indicated that the intervention reduced insomnia in employees with regular daytime work. The results also suggest that workplace-based group CBT may improve sleep in employees with primary insomnia if not concomitant with high burnout scores.

**Electronic supplementary material:**

The online version of this article (10.1007/s00420-018-1291-x) contains supplementary material, which is available to authorized users.

## Introduction

Sleep problems are very common in the working population (Kessler et al. 2011; Lallukka et al. 2010) and negative daytime consequences are often reported in association with sleep complaints (Cooper and Dewe 2008; Ford et al. 2011; Haaramo et al. 2012; Hui and Grandner 2015; Kyle et al. 2013; Rosekind et al. 2010; Riemann and Voderholzer 2003; Shekleton et al. 2010; Söderström et al. 2012). This implies a considerable economic burden both for the individual, the employer and society (Blom et al. 2015; Daley et al. 2009; Sivertsen et al. 2009; Walsh 2004). Consequently, cost-effective, evidence-based, and easy to use interventions are motivated to treat incipient sleep problems and prevent development of more severe and chronic sleep disorders among employees.

A group intervention for insomnia administered at the workplace would meet these requirements. Two recent meta-analyses (Koffel et al. 2015; Navarro-Bravo et al. 2015) confirm the efficiency of group CBT for insomnia on symptoms assessed through validated scales, and sleep parameters such as sleep efficiency measured through diaries. However, among the 13 randomized controlled trials included, none had focused specifically on working individuals and the interventions were mainly conducted in clinical settings.

Insomnia is commonly present together with other conditions, such as anxiety, depression and burnout (Bélanger et al. 2004; Ekstedt et al. 2009; Johnson et al. 2006; Lustberg and Reynolds 2000; Riemann 2007; Roth and Drake 2004; Söderström et al. 2012). Earlier studies have found that CBT for insomnia is effective even in the presence of anxiety and depression (Lichstein et al. 2010; Manber et al. 2008; Rybarczyk et al. 2009; Smith et al. 2005; Stepanski and Rybarczyk 2006) also when delivered in group settings (Blom et al. 2015; Belleville et al. 2011; Edinger et al. 2009; Germain et al. 2006; Järnefelt et al. 2014; Okajima et al. 2011; Ye et al. 2015). However, there is a lack of studies investigating the effect of CBT for insomnia comorbid with burnout or stress-related exhaustion.

Two recent systematic reviews based on prospective studies show a relationship between high psychosocial work stress and an increased risk of sleep disturbances (Linton et al. 2015; Van Laethem et al. 2013). Moreover, studies using physiological sleep measurements have shown that in particular high job burnout interferes with sleep architecture and causes sleep fragmentation (Ekstedt et al. 2009). Despite the well-defined link between work-related stress and sleep problems, this is to our knowledge the first randomized controlled study to evaluate the efficacy of a group CBT-intervention for insomnia in a working population, conducted at the workplace.

### Aim

The present study aimed to investigate if a workplace-based intervention could improve sleep among employees with moderate insomnia symptoms. The study was made through evaluation of a group CBT-intervention for insomnia (called ‘sleep school’) by means of a randomized controlled trial. Sleep was evaluated both objectively, through actigraphy and subjectively, through diary ratings and questionnaires. A participation in the group intervention during work hours was expected to reduce insomnia symptoms and improve sleep parameters such as subjective sleep quality and quantitative sleep efficiency. Moreover, an explorative aim was to evaluate the impact of level of burnout scores at baseline on the effect of the intervention.

## Methods

### Design

Twenty workplaces in the retail sector were invited to participate in the study. Employees would then be able to participate in a group CBT-program free of charge, at their own head office during working hours over a period of 3 months. They were informed that the study comprised randomization into an intervention group or a control group and that the control group had to wait at least 6 months before participating in the group intervention. Participants received two cinema tickets after completing measurements at baseline as well as after having fulfilled all three periods of data collection.

Two workplaces were interested in participating in the study and the subjects included were employees from offices, stores, warehouses and logistics. Applicants were informed that participation was voluntary and could at any time be withdrawn. The study was approved by the regional ethical committee in Stockholm (No. 2013/2043-31/2) and informed consent was obtained from all included individuals.

There were two inclusion criteria; employees (1) should work at least 75% of full time (corresponding to at least 30 weekly working hours), and (2) report regular (at least four to five times a week) sleep problems. Participants were excluded if they reported an active substance abuse problem or a diagnosed severe mental illness.

A psychologist made the screening via a short web-based questionnaire on insomnia symptoms (e.g. “How many times a week do you… (1) Have difficulties falling asleep? (2) Wake up several times during the night with difficulties going back to sleep? (3) Get daytime consequences due to poor sleep?”). In case of self-reported sleep apnea (“Do you suffer at least 2–3 times a week from sleep apnea; gasping or snoring during sleep?”), applicants were recommended to visit the occupational health care to get evaluation and advice for treatment. In total, 72 out of the 73 applicants met the criteria and were then offered to participate in the group CBT-program. Randomization was made through the research randomizer tool on http://www.randomizer.org.

### Study sample

Out of the 72 included individuals, 2 individuals chose to refrain before randomization. After the randomization, another 6 out of the 70 remaining participants chose to refrain (4 from the intervention group and 2 from the control group). They declared they had too much to do, or were going on leave of absence, or were on sick-leave or were to change workplace.

**Fig. 1:**
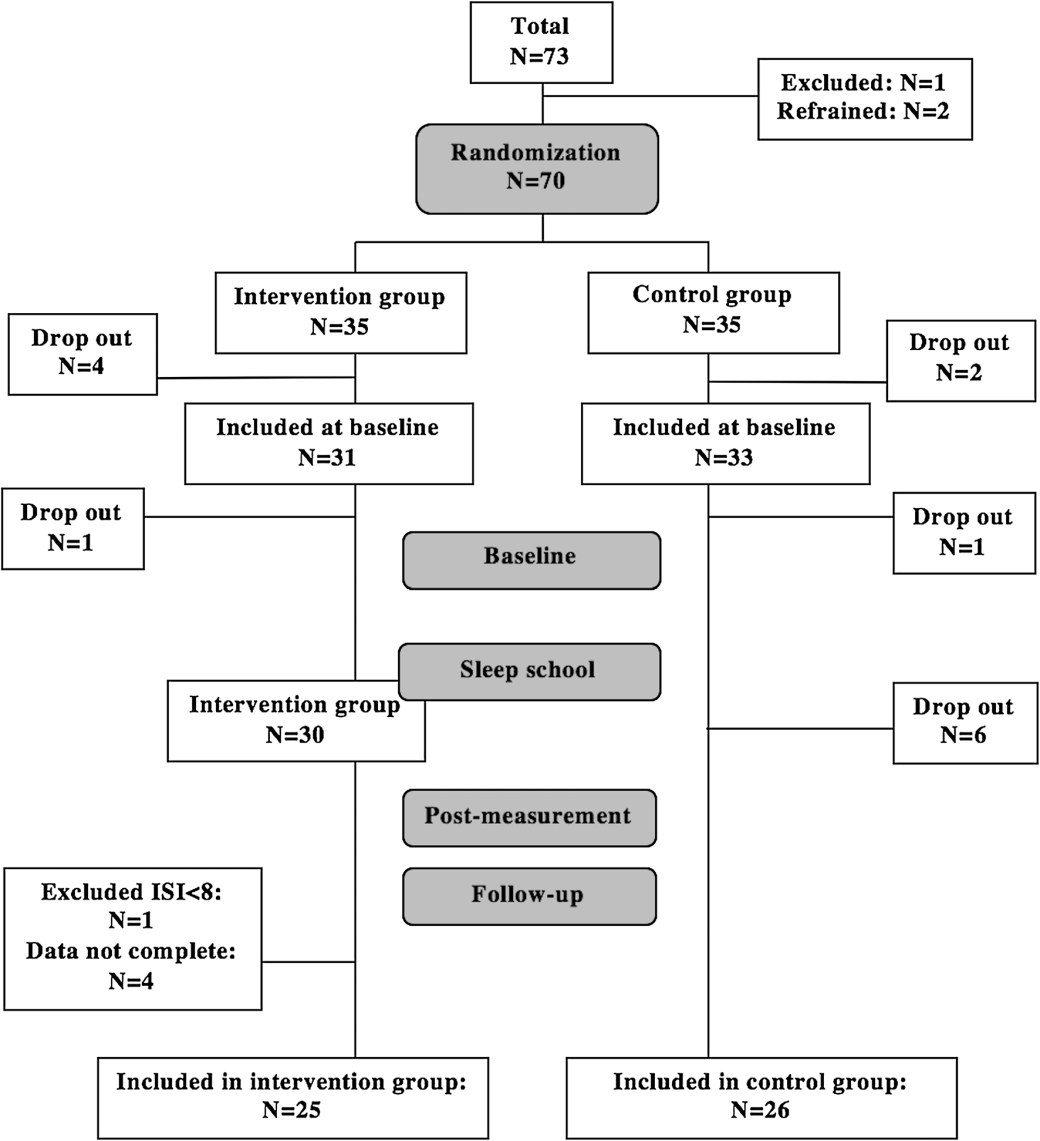
Flow chart over drop-outs and the number of participants fulfilling data collections

Altogether 64 individuals were invited to participate in the baseline measurement. However, two participants dropped out before the baseline measurement, and another six from the control group chose to refrain after the first measurement period because of sick-leave, time pressure or diminishing interest in participating in the study. Four individuals from the intervention group were excluded from the statistical analyses, since only data from participants who fulfilled measurements at baseline and either the post-measurements or the follow-up measurements, or both, were used. Furthermore, one participant in the intervention group, who reported a value of ISI at baseline lower than 8, was excluded from the analyses, since that indicated no presence of clinical insomnia. This resulted in a sample-size of *N* = 51 (see Fig. 1).

Participants worked in four different working areas; store, office, warehouse or logistics although two-thirds worked in offices. Those working at the office had regular working hours, while those working in stores often had irregular and flexible work time arrangements. Employees within warehouse and logistics typically work in regular shifts (but no night shifts). Background parameters are presented in Table 1. The age range was 22–60 years, 63% were women and about one-third of the participants reported they had children living at home.

**Table 1:** Groups at baseline; demographic data and type of employment

	Intervention group, *N* = 25	Control group, *N* = 26	Group differences^a^, *p* value
Age, mean (years)	*m* = 43.0 (*sd* = 9.3), range 28–60	*m* = 42.0 (*sd* = 8.8), range 22–58	0.684
Women	64%	62%	0.857
Education; university	52%	42%	0.753
Children 0–18 years old living at home	33%^b^	35%	0.871
Working full time	91%^c^	77%	0.200
Married/cohabitant	72%	69%	0.830
Employment: store	8%	8%	0.968
Office	76%	65%	0.410
Warehouse	12%	27%	0.284
Logistics	4%	0%	0.308
Manager	12%	27%	0.184

*p* values of group differences

*m* mean, *sd* standard deviation

^a^*T* test for comparison of age. Chi-squared test for non-parametric comparisons

^b^One missing value

^c^Three missing values

The majority (73%) of the participants suffered from clinical levels of insomnia at baseline (ISI > 14), 60% showed high levels of burnout scores (SMBQ > 3.75), 42% showed clinical levels of anxiety (HADSa > 10) and 8% clinical levels of depression (HADSd > 10). See Table 2. Chi-squared tests showed that the groups did not differ at baseline (*p* > 0.156). Correlations between ISI, SMBQ, HADSd and HADSa at baseline are presented in Table 3, showing that there is a significant and positive correlation between insomnia (according to ISI), high burnout scores (according to SMBQ) and anxiety (according to HADS). Attrition analyses were made to investigate selection bias; independent *t* tests did not show any significant differences between completers (participants having completed baseline measurement as well as the post- and/or follow-up measurement; *N* = 51) and non-completers (*N* = 4 in intervention group, *N* = 6 in control group) in terms of gender, age or level of ISI at baseline (*p* > 0.669).

**Table 2:** Percentage of subjects with high level of burnout scores and clinical levels of insomnia, depression and anxiety at baseline

	Intervention group, *N* = 25	Control group, *N* = 26	Group differences^a^, *p* value
Clinical insomnia (ISI > 14)	68%	77%	0.480
High level of burnout (SMBQ ≥ 3.75p)	54%^b^	67%^c^	0.381
Depression (HADSd > 10p)	4%	12%^b^	0.302
Anxiety (HADSa > 10p)	32%	52%^b^	0.156

*p* values of group differences

*ISI* Insomnia Severity Index, *SMBQ* Shirom–Melamed Burnout Questionnaire, *HADS* Hospital Anxiety and Depression Scale

^a^Evaluated through Chi-squared tests

^b^One missing value

^c^Two missing values

**Table 3:** Correlations (*r*) between baseline levels of insomnia and mental health for all participants

	ISI	HADSd	HADSa
HADSd	0.20 (0.40*)	–	–
HADSa	0.33* (0.57*)	0.55** (0.64**)	–
SMBQ	0.30* (0.50*)	0.64** (0.70**)	0.77** (0.84**)

Values for intervention group in parentheses

*ISI* Insomnia Severity Index, *SMBQ* Shirom–Melamed Burnout Questionnaire, *HADS* Hospital Anxiety and Depression Scale

**Correlation (*r*) is significant at the 0.01 level (2-tailed)

*Correlation (*r*) is significant at the 0.05 level (2-tailed)

### Procedure

Baseline measurements were made before the start of the intervention. Post-measurements were made approximately 3 months later, directly after the intervention was finished, and follow-up measurements another 3 months later. When all measurement periods were completed, the control group started their participation in the group CBT-program. Consequently, no data were collected after this point in time, except for an evaluation questionnaire which was distributed after participation in the program. During data collections, participants filled out a sleep-and-wake diary and wore a wrist-actigraph for 10 days. They also filled out a web-based questionnaire. See time-line in supplementary figure S1.

### Measurements

The questionnaire, which was distributed at each data collection period, included questions on demographic factors, working parameters as well as questions on sleep and stress levels. Insomnia symptoms were measured through the Insomnia Severity Index (ISI), which has robust psychometric properties (Bastien et al. 2001), even when administrated online (Thorndike et al. 2011) and in the presence of comorbid insomnia (Geiger-Brown et al. 2015). The ratings of the seven questions on perceived sleep disturbances during the two last weeks in ISI, given on a 5-graded scale (0 good sleep–4 very poor sleep) were summarized. Values between 0 and 7 correspond to no presence of insomnia, 8–14 subthreshold clinical insomnia, 15–21 presence of clinical insomnia and 22–28 severe clinical insomnia (Bastien et al. 2001).

Burnout scores were measured through the Shirom–Melamed Burnout Questionnaire (SMBQ; Melamed et al. 1992). Symptoms of burnout were rated on a 7-graded scale (1 almost never–7 almost always) according to the question “Please indicate to what extent these feelings usually occur”. The mean of the 22 questions gives a measure of burnout with values of ≥ 3.75p indicating high level of burnout scores (Grossi et al. 2003).

Depression and anxiety were evaluated through the Hospital Anxiety and Depression Scale (HADS; Zigmond and Snaith 1983; Sullivan et al. 1993). The seven items of each scale (anxiety and depression respectively) on experiences during the last week were rated on a 4-graded scale (from 0 to 3), giving global scorings of 0–7 non-cases, 8–10 possible cases and 11–21 probable cases.

During data collections, each morning, participants were asked to answer a modified and shorter version of the Karolinska Sleep Diary (KSD; Åkerstedt et al. 1997) that included questions on stress or worries at bedtime (1 very worried, aroused–5 very calm/relaxed), subjective sleep quality (How did you sleep?; 1 very poorly–5 very well) and non-refreshing sleep (Do you feel refreshed?; 1 not at all–5 completely).

In addition, participants wore a wrist-actigraph (Actiware Spectrum Pro by Philips Respironics) day and night, on the non-dominant hand, during the measurement period. Participants were asked to push an event button at lights out in the evening and at final wake-up time in the morning. Movements are measured though a sensitive accelerometer, which makes it possible to classify if the wearer has slept or not, based on the amount of movements per minute. Thereby, the length of the sleep period and the proportion of time awake during the night (sleep efficiency in percent), could be calculated. Sleep length refers to the time between sleep onset and wake-up. Sleep efficiency is based on calculations of the amount of time awake during sleep time. High efficiency corresponds to low amounts of time awake. Scorings and calculations were made in ActiWare Software version 6.0.2 (http://www.actigraphy.com/solutions/actiware/). Actigraphy is a well-established and validated objective method, complementing the individual’s own ratings in the diary (Sadeh 2011). Mean values were calculated on actigraphy data as well as on diary data for each measurement period.

ISI has been extensively used in CBT-research (Blom et al. 2015; Geiger-Brown et al. 2015; Ho et al. 2015; Navarro-Bravo et al. 2015; Seyffert et al. 2016) and constitutes the primary sleep outcome in the present study. However, actigraphy was needed to measure outcomes related to quantity of sleep, such as sleep duration and sleep efficiency. Furthermore, diary measurement of sleep provides psychometric strengths in terms of low risk of recall bias and high reliability when evaluating progress in relation to an intervention (Bolger et al. 2003; Libman et al. 2000).

### Group CBT-intervention: procedure

The sleep school was a CBT-based program involving both theory and practice, developed and led by a trained, certified clinical psychologist. The program was carried out in groups (maximum eight participants in each group) and included five sessions (of 2 h) during a period of approximately 3 months. The aim was to increase participants’ knowledge about sleep and to provide practical tools to improve sleep and to decrease stress and worries about sleep.

All sessions included a psycho-educative lecture and group discussions. At the end of each session, participants were encouraged to choose a personal homework related to the topic of the session. The program involved five sections (one section per session): (1) basic knowledge of sleep, circadian rhythm and sleep regulation, (2) lifestyle factors, including evening routines, physical activity, alcohol and caffeine use, (3) sleep schedules, sleep restriction and stimulus control, (4) stress and daily balance of activity and relaxation/rest, and (5) mindfulness and acceptance strategies. Absence from sessions were recouped through instructions via e-mail from the group-leader together with the digital presentation from the session and assigned homework.

### Evaluation questionnaires

After completing the group CBT-intervention, participants answered an evaluation questionnaire on how they had experienced the program and how much they had been engaged; e.g. “Has the sleep school taken a lot of your time? Has it been effective? Has it been difficult?” (0 I do not agree–4 I completely agree) or “How many sessions have you participated in?” (0, 1, 2, 3, 4 or 5) “Have you recouped the sessions you missed?” (Yes/No) and “I would recommend participation in a sleep school to employees within retail” (Yes/No/Maybe).

Moreover, the (waiting list) control group answered questions to determine if some of the participants had underwent any other type of intervention against their sleep problems during the period of measurements. This was made to ensure their function as a control group against the intervention group.

### Statistical analyses

The statistical analyses were based on multilevel mixed modelling. The model included the outcome variable (e.g. ISI scores or mean value of sleep duration) and the fixed effects of the between-group factor Group (Intervention vs Control; level 2), the within-group factor Time (Baseline, Post-measurement and Follow-up; level 1) and the interaction between Group and Time. Because of the nested data over time, the model was fitted by modelling the autocorrelation. Additional analyses were performed; two sensitivity analyses and item analyses of the seven items included in ISI. These results are presented in a supplement (Table S1 to S5). The first sensitivity analysis was made by excluding employees in warehouse and logistics from the sample (four participants from the intervention group and seven from the control group), since they were typically working shifts. The second one was made by including only the 17 completers in the model.

Evaluation of the moderating effect of burnout scores at baseline (SMBQbl) was made by adding a second between-group factor; SMBQbl (high vs low; cut-off 3,75) into the model, resulting in several two-way interactions and a three-way interaction of Group × Time × SMBQbl. The three-way interaction reveals if there is an effect on sleep that depends on both group affiliation (intervention vs control) and level of burnout score (high vs low) over time. Furthermore, post hoc analyses were made only on the intervention group through a model where the between-group factor was SMBQ-bl (high vs low) and the within-group factor was time. A two-tailed alpha-level of 0.05 was used when testing for statistical significance. Descriptives, *t* tests and Chi-squared tests were carried out in SPSS 24, whereas multilevel analyses were made in STATA 14.

## Results

The proportions of participants in the intervention group with values over the threshold for clinical insomnia dropped from 68% at baseline to 33% after the intervention and were 35% 3 months later. The corresponding data for the control group were 77% at baseline, 65% post-intervention and 62% at follow-up. Using diagnostic criteria, 53% in the intervention group were in remission (ISI ≥ 15 at baseline and < 15 at follow-up), compared to 20% in the control group. Contrast scores were calculated separately by group and time period, and it was found that the intervention group improved significantly on ISI scores between baseline and post-measurements (− 2.534; *p* = 0.001), but not between post-measurements and follow-up. Thus, a significant within-group effect on insomnia was observed. The control group increased their sleep length (as measured with actigraphy) between baseline and post-measurements (Contrast = 0.258; *p* = 0.042) and they improved on ISI scores between post-measurement and follow-up (Contrast = − 1.530; *p* = 0.045). No other significant changes were found over time.

The multilevel mixed model showed no difference between groups over time in the degree of insomnia, nor in the sleep parameters as measured with sleep diary or with actigraphy. Mean values are presented in Table 4 and interaction effects in Table 5.

**Table 4:** Average values for both groups at the three measurement points

		Baseline		Post-measure		Follow-up	
Questionnarie data	Group	*m*	*sd*	*m*	*sd*	*m*	*sd*
ISI—insomnia (0 good sleep–28 poor sleep)	Intervention	16.04	3.9	13.58	6.0	12.96	5.6
	Control	16.42	4.4	16.69	4.8	15.43	3.8
SMBQ—burnout (1 low–6 high burnout)	Intervention	3.93	1.3	3.83	1.3	3.89	1.5
	Control	4.15	1.0	4.09	1.2	3.93	1.2
**Diary data** ^a^							
Mean subjective sleep quality (1 very poor–5 very good)	Intervention	3.01	0.6	3.08	0.7	3.14	0.7
	Control	2.98	0.5	3.06	0.7	3.11	4.1
Mean non-refreshing sleep (1 non refreshed–5 completely)	Intervention	2.67	0.6	2.85	0.6	2.75	0.8
	Control	2.48	0.6	2.51	0.6	2.55	0.5
Stress/worries at bedtime (1 very worried–5 very calm)	Intervention	3.55	0.5	3.60	0.6	3.47	0.6
	Control	3.28	0.4	3.17	0.4	3.25	0.4
**Actigraphy data**							
Sleep length (h:mm)	Intervention	6:40	0:30	6:43	0:30	6:42	0:30
	Control	6:48	0:54	7:02	0:36	6:48	0:30
Sleep efficiency (%)	Intervention	88.0	3.6	87.5	5.8	88.5	2.7
	Control	86.9	6.9	87.1	4.5	86.1	3.6

*ISI* Insomnia Severity Index, *SMBQ* Shirom–Melamed Burnout Questionnaire, *HADS* Hospital Anxiety and Depression Scale, *m* mean, *sd* standard deviation

^a^Data is based on 10 days of measurement, including on average 7.4 workdays at baseline 6.6 workdays at post-measurement and 5.9 workdays at follow-up

**Table 5:** Results of the group by time interaction from the multilevel mixed model analyses

Questionnaire data	Estimate	SE	*z*	*P* > |*z*|	95% CI	
ISI—insomnia (0 good sleep–28 poor sleep)	− 1.050	0.632	− 1.65	0.098	− 2.285	0.193
SMBQ—burnout (1 low–6 high burnout)	0.010	0.138	0.08	0.940	− 0.261	0.282
**Diary data**						
Mean subjective sleep quality (1 very poor–5 very good)	0.006	0.078	0.07	0.943	− 0.148	0.159
Mean non-refreshing sleep (1 non refreshed–5 completely)	0.040	0.088	0.45	0.649	− 0.133	0.213
Stress/worries at bedtime (1 very worried–5 very calm)	− 0.030	0.052	− 0.56	0.575	− 0.132	0.073
**Actigraphy data**						
Total sleep time (h)	− 0.031	0.104	− 0.30	0.766	− 0.234	0.173
Sleep efficiency (%)	0.321	1.011	0.32	0.751	− 1.66	2.302

All three data collection periods are included in the model

*ISI* Insomnia Severity Index, *SMBQ* Shirom–Melamed Burnout Questionnaire, *SE* standard error, *CI* confidence interval

Sensitivity analyses were made by excluding shift workers. A significant interaction effect between groups over time was observed in this analysis, with a decrease in ISI for the intervention group (Estimate = − 1.319, *p* = 0.044, CI = − 2.600 to − 0.038; see Tables S1 and S2 in supplement). Item analyses of the seven items constituting ISI showed two significant interaction effects of group over time; dissatisfaction with current sleep (*p* = 0.032) and experience sleep problems as disturbing (*p* = 0.045). These two components of insomnia significantly improved more over time in the intervention group compared to the control group (see Tables S3 and S4 in supplement).

### Exploring the moderating effect of burnout scores at baseline

**Fig. 2:**
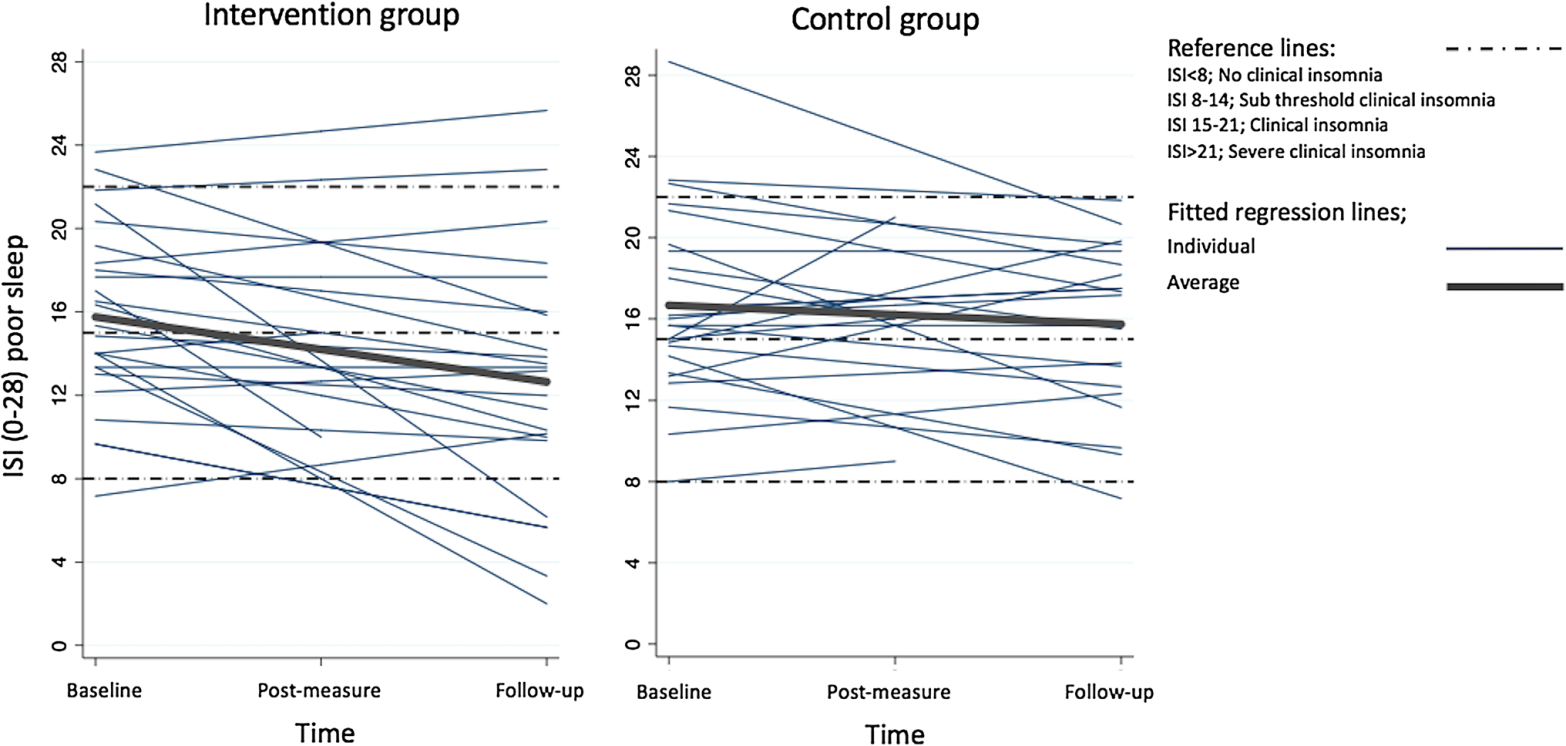
Spaghetti plot of development of Insomnia Severity Index (ISI) over time

There is an increasing variation in ISI with time (see distribution measurements in Table 4). Spaghetti plots (Fig. 2) illustrate these individual differences in relation to the intervention. Indeed, when adding SMBQbl as an additional factor in the model it was shown that burnout scores significantly moderated the improvement of ISI. This was indicated by a significant three-way interaction; Group × Time × SMBQbl (estimate 3.28; *p* = 0.009; CI 0.818–5.746). There were no differences between the intervention group and the control group in the baseline-levels of burnout scores (*t* = − 0.51, *p* = 0.612).

**Fig. 3:**
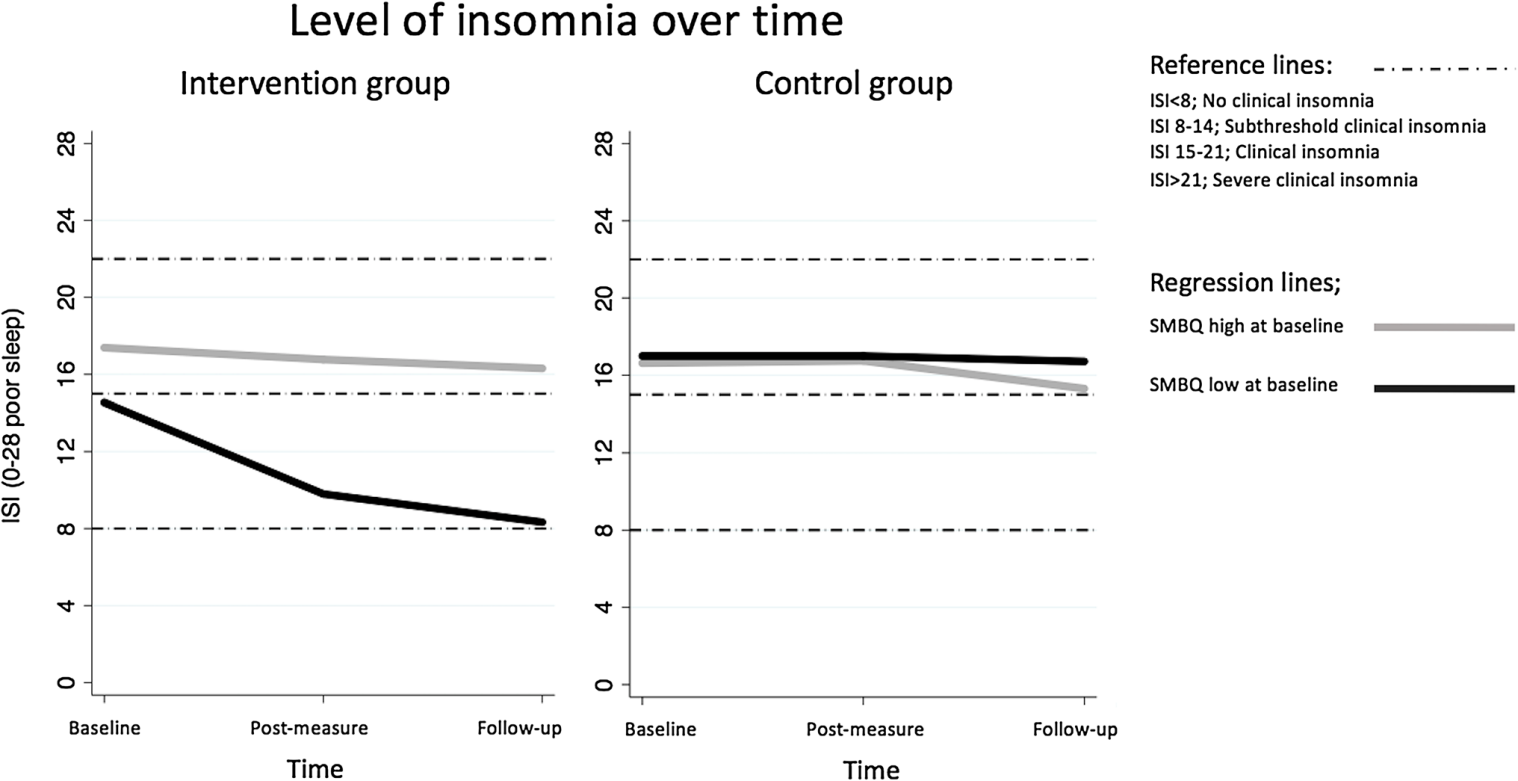
Presentation of the intervention group and the control group divided in high and low levels of burnout scores at baseline (threshold value of SMBQ ≥ 3.75p; Shirom–Melamed Burnout Questionnaire), as a function of insomnia over time (high values of ISI = poor sleep; Insomnia Severity Index) as a dependent variable

Further analyses were made on the intervention group only, which was divided into two groups based on the value of SMBQbl (*N*_high_ = 13, *N*_low_ = 11; cut-off 3.75). A significant interaction effect of SMBQbl × Time (Estimate 2.51; *p* = 0.005; CI 0.770–4.244) showed that participants with low levels of burnout scores at baseline significantly improved on insomnia over time, whereas participants with high burnout scores at baseline did not (see Fig. 3). The effects on other sleep parameters measured with diary or actigraphy were not moderated by the level of burnout scores at baseline (*p* = 0.129–0.409). A corresponding post hoc analysis on the control group revealed no differences between participants with high or low levels of burnout scores over time (see Fig. 3).

### Participants’ evaluation of the intervention

Altogether 36 out of 51 participants from both intervention group and control group completed the evaluation questionnaire after having participated in the group CBT-program. The results showed that 94% felt they had been helped by the intervention and that 91% would recommend the program to other employees within their organization. Participants were relatively satisfied with the structure of the program (86% answered “3” or “4” on a scale ranging from 0 I do not agree to 4 I completely agree) and a large part had the confidence in being able to handle their sleep problems in the future (86%). Half of the participants thought they were more well-rested in the morning (54%) and more alert during the day (54%) after having participated in the program. The majority (76%) felt calmer at bedtime.

### Adherence to the group intervention

In the intervention group, 17 out of the 22 participants who filled out the evaluation questionnaire were considered as having fully participated in the program; meaning they had participated in at least three sessions and in case of absence they had actively recouped the session. Sensitivity analyses, including only the 17 completers in the model, showed no differences in significant interaction effects compared to the original analyses (see Table S5 in supplement). Analyses through *t* tests showed no significant differences between completers and non-completers in level of burnout scores (*t* = − 1.08, *p* = 0.295, CI − 1.86–0.59) or insomnia symptoms at baseline (*t* = − 0.92, *p* = 0.367, CI − 2.01–6.06).

## Discussion

The hypothesis that a group CBT-program for insomnia in a workplace setting would lead to decreased levels of insomnia symptoms compared to a control group was partially confirmed. For the full sample, there was no improvement in insomnia based on ISI for the intervention group compared to the control group. There were no effects on sleep parameters such as subjective sleep quality, sleep length and sleep efficiency, measured through diary or actigraphy. However, within-group effects on insomnia symptoms were observed in the intervention group, as ISI scores decreased between baseline and follow-up. Moreover, when shift workers were excluded from the sample, a significant interaction effect between groups over time was observed through reductions of ISI scores in the intervention group compared to the control group.

These results are partially in line with previous studies on group CBT for insomnia, showing positive effects on insomnia symptoms assessed both through validated scales (e.g. ISI) and diaries, but no effects on sleep duration (Navarro-Bravo et al. 2015). Similarly, the meta-analysis by Koffel et al. (2015) found small effects on insomnia symptoms such as sleep efficiency and only within-group effects on ISI scores (*d* = − 0.70), sleep length and sleep quality.

The fact that there was no significant effect on sleep duration in the present study could be explained by one of the methods applied during the second half of the program; sleep restriction, where time in bed is diminished to enhance sleep efficiency. However, both sleep length and sleep efficiency, objectively measured with actigraphy, was considered being relatively good at baseline (see Table 3), leading to expectations of small observed effects in these sleep parameters. This could further explain the absence of significant effect on sleep efficiency.

Moreover, it should be noted that the effects of a CBT-program for insomnia might occur later in time, once the participants have actually implemented the tools and tested the different methods. In the meta-analysis by Koffel and colleagues (2015), patients continued to improve on total sleep time and sleep quality even after the group CBT-program had ended. Consequently, positive results on sleep might appear later than 3 months after completion of the group CBT-intervention. However, using a waiting list control group, a follow-up period longer than 3 months was not feasible. Importantly, improvements on ISI scores in the intervention group were found between baseline and post-measurement and not between post-measurement and follow-up in the present study.

Koffel et al. (2015) also found that increased total time spent in group sessions had better effect on sleep. The number of sessions in the included studies varied between 4 and 8, and each session was 60–120 min. In this present study, the program was relatively short with the ulterior motive to reduce time expenditure during work hours.

Interestingly, the item analyses of ISI showed no effects on the items measuring disturbed sleep, but positive effects in the two components ‘dissatisfaction with current sleep’ and ‘experiencing sleep problems as disturbing’. This indicates that the group CBT-intervention somehow influenced participants’ attitude to their sleep problems, but not their sleep disturbance. Notably, insomnia symptoms were moderate at baseline and larger effects might have been seen in a population with more severe insomnia symptoms.

### The moderating effect of level of burnout scores at baseline

A moderating effect of level of burnout scores at baseline was found, showing that there was a positive effect of the intervention on insomnia symptoms for individuals who scored low levels of burnout at baseline. Earlier studies have found group CBT for insomnia to be effective despite comorbid anxiety and depression (Blom et al. 2015; Lichtstein et al. 2010; Rybarczyk et al. 2009; Smith et al. 2005; Stepanski and Rybarczyk 2006), whereas in this study it was shown that comorbid burnout interfered with the effect of the intervention. The moderating effect of level of burnout scores at baseline is an interesting finding since no other study has investigated treatment efficiency related to stress levels in a workplace setting, even though sleep and stress are so closely related (Armon et al. 2008; Ekstedt et al. 2009; Linton et al. 2015; Van Laethem et al. 2013).

When suffering from high burnout levels, a group CBT-program at the workplace involving both presences during work hours, reading and other homework tasks might be too demanding. Another explanation could be that higher burnout scores relates to decreased ability to benefit and learn from the intervention, despite the level of effort (Vogel and Schwabe 2016).

## Strengths and limitations

The program was highly appreciated by the participants; almost all participants rated it as helpful and would recommend the program to other employees. Data-collections comprised several complementary and reliable instruments; subjective diary data and questionnaires as well as objective actigraphy, which provide well-founded results. The possible benefits or weaknesses of a workplace setting for such a sleep intervention program are not evaluated in this study. However, by locating the sessions at the workplace, the program is easily accessible for the participants (even though some employees needed to travel from the warehouse or the store to the main office) and it might provide a feeling of support from the workplace, which may increase motivation to participate in the treatment. Moreover, employees from the same workplace in a group setting can discuss potential shortcomings of their workplace and how these problems can be solved. The participants can also support and encourage each other to complete the homework related to the sessions and the measurements used for evaluating the program. Possible downsides of a workplace setting could be that participants do not feel comfortable talking openly about their problems together with their colleagues. In large organizations, as in the present study, this problem is however partly reduced. Moreover, a participation in the program may increase the workload unless job demands are not decreased during the days when they had a treatment session.

The main limitation of this study is that participant attrition led to reduced statistical power. The a-priori power calculation indicated that 64 participants (32 in intervention group and 32 in control group) would be the threshold for ensuring a large effect size of sleep-improvement. The present study included 51 participants.

Beside the limited statistical power, there are some additional limitations in this study that should be pointed out; first, the wide inclusion criteria lead to heterogeneity of the participants in terms of degree of insomnia, comorbidity and working conditions (e.g. employees working daytime vs shift workers). Importantly, the sensitivity analyses, by excluding employees working shifts, showed a significant interaction effect between groups over time on the level of insomnia symptoms. This suggests that group CBT for insomnia might need to be adapted to the specific sleep problems related to work shifts, such as having frequent sleep restriction and irregular sleep timing.

Moreover, using a waiting list control group in treatment studies might entail certain downsides. In this study, control questions revealed that almost half of the individuals in the control group had found other ways of handling their sleep problems during the measurement period. They had either seen a physician, tried a self-help program or made structural changes to overcome their problems. Furthermore, when having a waiting list control group at the same workplace as the intervention group, colleagues from the different groups might interact during the intervention. If the participants in the study group discussed the content of the intervention sessions, the control group may get tools and recommendations of how to improve their sleep despite a lack of formal treatment. For example, in this present study, the control group increased their sleep length and improved on insomnia symptoms during the measurement periods.

Finally, five participants in the intervention group were considered being non-completers; one participant only attended two sessions out of five and four participants did not recoup the one or two sessions they were absent. This might have affected the efficacy and the outcome of the program (Matthews et al. 2013).

## Practical implications and future research

Given the results of this study, it might be of interest to take the level of burnout symptoms (operationalized as high burnout scores) into account when implementing a program for sleep problems in a workplace setting. Levels of burnout scores should preferably be evaluated before participation to enable a customization of the program. Such a customized program could, for example, be internet-based or available via an app (Bostock et al. 2016; Blom et al. 2015), allowing participants to work at their own pace. Subjects with more severe stress symptoms or high burnout scores should receive adequate help for these symptoms. Results should preferably be generalized to large companies, since we do not know whether size of the company may influence the possibility to participate in the sessions during normal working hours. Notably, it is very important to point out that the sleep program should not replace organization interventions aiming to improve, for example, work scheduling and the balance between work load and recovery.

Future studies should investigate possible benefits of a workplace setting as well as changes in work ability and productivity in relation to such a program.

## Conclusion

We conclude that a group CBT-intervention for insomnia in a workplace setting did not improve sleep for the investigated group as a whole over a 3-month period when compared to a waiting list control group. However, within-group changes, and analyses of changes in subgroups were promising, showing that the intervention reduced insomnia in working subjects with low levels of concurrent burnout scores and employees having daytime work. These findings indicate that a workplace-based group CBT-intervention for insomnia might be a feasible method to treat sleep problems and prevent development of more severe and chronic sleep disorders. However, the program employed in this present study needs to be developed and further evaluated in employees outside the retail sector.

## Electronic supplementary material

Below is the link to the electronic supplementary material.

Supplementary material 1 (DOCX 116 KB)
