# Supplementary material for: A randomized controlled intervention of workplace-based group cognitive behavioral therapy for insomnia
Source: Int Arch Occup Environ Health. 2018 Jan 31;91(4):413–24. doi: 10.1007/s00420-018-1291-x (PMC5908834; doi:10.1007/s00420-018-1291-x)
Supplement: Supplementary file 1 — Supplementary material 1 (DOCX 116 KB) [file 420_2018_1291_MOESM1_ESM.docx]

**Supplement**

**A randomized controlled intervention of workplace-based group cognitive behavioral therapy for insomnia**

**Figure S1.** Time-line of the procedure.

**
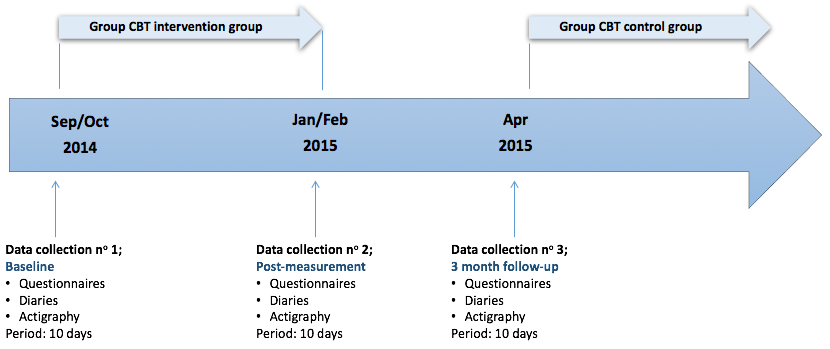
**

**Table S1.** Sensitivity analyses I; workers from warehouse and logistics (N=11) were excluded from the original sample. Results of the group by time interaction from the multilevel mixed model analyses. All three data collection periods are included in the model.

|  | Estimate | S.E.^c^ | z | P>\|z\| | 95% C.I.^d^ |
| --- | --- | --- | --- | --- | --- |

| *Questionnaire data* |  |  |  |  |  |  |
| --- | --- | --- | --- | --- | --- | --- |
| ISI^a^ – insomnia  (0 good sleep – 28 poor sleep) | -1.319 | 0.654 | -2.02 | 0.044* | -2.600 | -0.038 |
| SMBQ^b^ - burnout  (1 low – 6 high burnout) | 0.082 | 0.165 | 0.50 | 0.619 | -0.242 | 0.407 |

***Diary data***

| Mean subjective sleep quality  (1 very poor – 5 very good) | -0.028 | 0.087 | -0.33 | 0.744 | -0.199 | 0.142 |
| --- | --- | --- | --- | --- | --- | --- |
| Mean non-refreshing sleep  (1 non refreshed – 5 completely) | 0.028 | 0.088 | 0.32 | 0.747 | -0.144 | 0.201 |
| Stress/worries at bedtime  (1 very worried – 5 very calm) | -0.007 | 0.060 | -0.12 | 0.908 | -0.125 | 0.111 |

***Actigraphy data***

| Total sleep time  (hours) | -0.065 | 0.113 | -0.58 | 0.565 | -0.286 | 0.156 |
| --- | --- | --- | --- | --- | --- | --- |
| Sleep efficiency  (%) | 0.164 | 1.143 | 0.14 | 0.886 | -2.077 | 2.405 |

* Significant at the 0.05-level

a) ISI=Insomnia Severity Index

b) SMBQ=Shirom Melamed Burnout Questionnaire

c) S.E.=Standard Error

d) C.I.=Confidence Interval

**Table S2.** Mean values and standard deviations of ISI (significant in Sensitivity analyses I) for the different groups.

| Variable | Group | Baseline  m^a^ sd^b^ | | Post  m sd | | Follow-up  m sd | |
| --- | --- | --- | --- | --- | --- | --- | --- |
| ISI^a^ – insomnia  (0 good sleep – 28 poor sleep) | Intervention | 15.62 | 0.8 | 12.28 | 0.8 | 11.90 | 1.1 |
|  | Control | 16.21 | 1.0 | 16.32 | 1.0 | 15.33 | 1.0 |

1. m=mean
2. sd=standard deviation

**Table S3.** Item analyses of ISI; Results of the group by time interaction from the multilevel mixed model analyses. All three data collection periods are included in the model.

| Variables | Estimate | S.E.^b^ | z | P>\|z\| | 95% C.I.^c^ | |
| --- | --- | --- | --- | --- | --- | --- |
| ISI^a^ item 1  Seriousness Sleep onset  1 - 5 very serious | -0.023 | 0.131 | -0.18 | 0.859 | -0.281 | 0.234 |
| ISI item 2  Seriousness Awakenings  1 – 5 very serious | -0.181 | 0.154 | -1.18 | 0.238 | -0.482 | 0.120 |
| ISI item 3  Seriousness Wake up too early  1 – 5 very serious | -0.252 | 0.166 | -1.52 | 0.129 | -0.577 | 0.074 |
| ISI item 4  Dissatisfaction with current sleep  1 – 5 very dissatisfied | -0.302 | 0.141 | -2.15 | 0.032* | -0.578 | -0.026 |
| ISI item 5  Sleep problem is disturbing  1 – 5 very disturbing | -0.221 | 0.110 | -2.01 | 0.045* | -0.437 | -0.005 |
| ISI item 6  Other people notice my problem  1 – 5 very notable | 0.083 | 0.146 | 0.57 | 0.571 | -0.204 | 0.370 |
| ISI item 7  Worry about current sleep  1 – 5 very worried | -0.189 | 0.138 | -1.37 | 0.171 | -0.458 | 0.081 |

* Significant at 0.05-level

a) ISI=Insomnia Severity Index

b) S.E.=Standard Error

c) C.I.=Confidence Interval

**Table S4.** Mean values and standard deviations of ISI item four and five (significant in Item analyses of ISI) for the different groups.

| Variable | Group | Baseline  m^a^ sd^b^ | | Post  m sd | | Follow-up  m sd | |
| --- | --- | --- | --- | --- | --- | --- | --- |
| ISI item 4  Dissatisfaction with current sleep  (1 – 5 very dissatisfied) | Intervention | 4.12 | 0.1 | 3.42 | 0.2 | 3.43 | 0.2 |
|  | Control | 4.08 | 0.1 | 4.12 | 0.1 | 3.95 | 0.2 |
| ISI item 5  Sleep problem is disturbing  (1 – 5 very disturbing) | Intervention | 3.68 | 0.2 | 3.17 | 0.2 | 3.26 | 0.2 |
|  | Control | 3.73 | 0.2 | 3.69 | 0.2 | 3.76 | 0.2 |

1. m=mean
2. sd=standard deviation

**Table S5.** Sensitivity analyses II; only participants who reported they completed the group CBT-intervention (N=17) are included the model. Results of the group by time interaction from the multilevel mixed model analyses. All three data collection periods are included in the model.

|  | Estimate | S.E.^c^ | z | P>\|z\| | 95% C.I.^d^ |
| --- | --- | --- | --- | --- | --- |

| *Questionnaire data* |  |  |  |  |  |  |
| --- | --- | --- | --- | --- | --- | --- |
| ISI^a^ – insomnia  (0 good sleep – 28 poor sleep) | -1.030 | 0.702 | -1.47 | 0.142 | -2.407 | 0.347 |
|  |  |  |  |  |  |  |
| SMBQ^b^ - burnout  (1 low – 6 high burnout) | -0.034 | 0.158 | -0.22 | 0.830 | -0.344 | 0.276 |
|  |  |  |  |  |  |  |

***Diary data***

| Mean subjective sleep quality  (1 very poor – 5 very good) | -0.023 | 0.088 | -0.26 | 0.795 | -0.194 | 0.149 |
| --- | --- | --- | --- | --- | --- | --- |
| Mean non-refreshing sleep  (1 non refreshed – 5 completely) | 0.077 | 0.096 | 0.80 | 0.427 | -0.112 | 0.265 |
| Stress/worries at bedtime  (1 very worried – 5 very calm) | -0.027 | 0.055 | -0.49 | 0.621 | -0.136 | 0.081 |

***Actigraphy data***

| Total sleep time  (hours) | -0.063 | 0.118 | -0.54 | 0.591 | -0.294 | 0.168 |
| --- | --- | --- | --- | --- | --- | --- |
| Sleep efficiency  (%) | 0.381 | 1.166 | 0.33 | 0.744 | -1.905 | 2.667 |

a) ISI=Insomnia Severity Index

b) SMBQ=Shirom Melamed Burnout Questionnaire

c) S.E.=Standard Error

d) C.I.=Confidence Interval
